# A Perspective of the Cross-Tissue Interplay of Genetics, Epigenetics, and Transcriptomics, and Their Relation to Brain Based Phenotypes in Schizophrenia

**DOI:** 10.3389/fgene.2018.00343

**Published:** 2018-08-23

**Authors:** Jingyu Liu, Jiayu Chen, Nora Perrone-Bizzozero, Vince D. Calhoun

**Affiliations:** ^1^Mind Research Network, Lovelace Biomedical and Environmental Research Institute, Albuquerque, NM, United States; ^2^Department of Neurosciences, University of New Mexico, Albuquerque, NM, United States; ^3^Department of Electrical and Computer Engineering, University of New Mexico, Albuquerque, NM, United States

**Keywords:** cross-tissue, epigenetic, transcriptomic, brain based phenotype, schizophrenia

## Abstract

Genetic association studies of psychiatric disorders have provided unprecedented insight into disease risk profiles with high confidence. Yet, the next research challenge is how to translate this rich information into mechanisms of disease, and further help interventions and treatments. Given other comprehensive reviews elsewhere, here we want to discuss the research approaches that integrate information across various tissue types. Taking schizophrenia as an example, the tissues, cells, or organisms being investigated include postmortem brain tissues or neurons, peripheral blood and saliva, *in vivo* brain imaging, and *in vitro* cell lines, particularly human induced pluripotent stem cells (iPSC) and iPSC derived neurons. There is a wealth of information on the molecular signatures including genetics, epigenetics, and transcriptomics of various tissues, along with neuronal phenotypic measurements including neuronal morphometry and function, together with brain imaging and other techniques that provide data from various spatial temporal points of disease development. Through consistent or complementary processes across tissues, such as cross-tissue methylation quantitative trait loci (QTL) and expression QTL effects, systemic integration of such information holds the promise to put the pieces of puzzle together for a more complete view of schizophrenia disease pathogenesis.

## Background

Due to advances in genomic technology and world-wide collaborations in genotype–phenotype association studies, the past decade has witnessed exciting new discoveries regarding the genetic risk for various complex diseases or traits. Specifically for schizophrenia (SZ), a complex neuropsychiatric disorder, large scale genome-wide association studies (GWAS) have presented with high confidence an unprecedented genetic risk profile, indicating a high polygenic nature of the disease, with contributions of both common and rare mutations, and a surprisingly large proportion of risk related to non-coding chromosome regions (Stefansson et al., 2008; Bassett et al., 2010; Schizophrenia Working Group of the Psychiatric Genomics Consortium, 2014). In general the heritability of SZ is estimated to be around 80% (Cannon et al., 1998; Sullivan et al., 2003), and genetics play an important role in its etiology and pathophysiology. On the other hand, discordance between monozygotic twins also argues for the significant influence of environment and interaction between genetics and environment. To achieve a better understanding of the pathogenesis of SZ, identification of its genetic risk, although of high importance, is just a starting point. This viewpoint has prompted researchers to start the shift from genetic risks into more in-depth investigations toward causal mechanisms.

While several recent reviews have recapitulated new evidences for SZ etiology and pathophysiology from the perspective of epigenetics (Hoffmann et al., 2016; Swathy and Banerjee, 2017), transcriptomics (Lein et al., 2017), imaging genomics (Liu and Calhoun, 2014; Mufford et al., 2017), and others (Gandal et al., 2016; Birnbaum and Weinberger, 2017; Konopka, 2017; Owen and O’Donovan, 2017), here we want to emphasize the interplay among these domains, with a focus on approaches that integrate information across various tissue types. Psychiatric disorders in general all face the challenge of accessibility of the primary tissue of interest – the brain. Thus, alternative tissues have been investigated in hope that conserved or complementary mechanisms across tissue types could be identified to get further insights into the pathogenesis and pathophysiology of disease. Using SZ as an example, the tissues, cells, and organisms that are being investigated include postmortem brain tissues or neurons, peripheral blood and saliva, *in vivo* brain structure and function, and *in vitro* cell lines, particularly induced pluripotent stem cells (iPSC) and iPSC derived neurons (iPSNs). Different tissues or cells, in a broad sense, provide information at various spatial temporal points of disease development. Of particular interest here are the molecular signatures (e.g., genetics, epigenetics, and transcriptomics), neuronal activation, brain morphometry and activity. Integrating them systemically, ideally with information from proteomics and animal models, which is not included in this selective review, could help put the pieces of puzzle together toward a more complete view of SZ disease pathogenesis.

## Overview of Information Derived from Various Tissues

**FIGURE 1:**
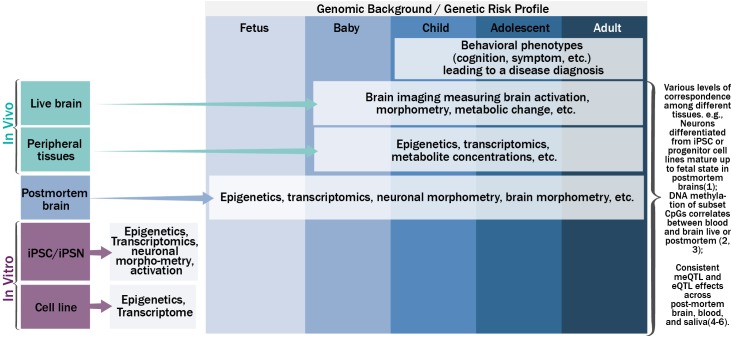
The schematic relationship among genetics, epigenetics, transcriptomics, and phenotypic manifestations on various tissues along the life span.

**Figure 1** shows the schematic relationship among genetics, epigenetics, and transcriptomics from various tissues along the life span, and their potential phenotypic manifestations. Under a specific genomic background, carrying a risk profile, causal genetic mutations may lead to changes in gene expression (i.e., the transcriptome) directly, such as mutations residing in the gene coding regions that change mRNA sequence; or indirectly, through altering or interacting with epigenetic regulation mechanisms to up- or down-regulate gene expression or produce different splicing isoforms. Mutations in the transcriptome of a cell could change the amino acid sequence of protein, protein structure, or protein shape, and further the biology of a cell (e.g., morphometry and function). A population of such cells could lead to anomalies in the function of an organism. While this simplified version of a causal pathway for genetic mutations could occur in various types of tissues in a tissue specific and dynamic fashion, consistent or complementary processes across tissue types are also plausible to some extent [for more complete review on translating genetics into disease mechanisms can be seen elsewhere (Gandal et al., 2016)]. For example, neurons differentiated from neural progenitor cell lines or iPSC *in vitro* have demonstrated high preservation of neural development processes observed by transcriptome analyses of postmortem brains at different stages of development (Stein et al., 2014). Blood-brain DNA methylation correlations, where methylation status in blood can explain large percentage (>50%) of variance of methylation in the brain, has been reported for a set of cytosine-phosphate-guanine dinucleotides (CpG) for both live brains and postmortem brains (Hannon et al., 2015; Walton et al., 2016). DNA methylation age can be reliably estimated using a variety of tissues from the same set of CpG sites (Horvath, 2013). Similarly, gene expression levels from certain genes showed correspondence between blood and brain (Glatt et al., 2005). DNA methylation quantitative trait loci (meQTL), and gene expression quantitative trait loci (eQTL) have repeatedly shown consistency across various tissues (Torres et al., 2014; Andrews et al., 2017; Lin et al., 2018b), where SNPs regulate epigenetics and/or transcriptomics in a consistent or complementary fashion across multiple tissue types.

In parallel, the dynamic nature of epigenetics, transcriptomics, and cellular and organism phenotypes warrants the inclusion of a temporal dimension in the disease pathogenesis model. The temporal dynamics of molecular signatures of brain tissues could be captured by examining postmortem brains from the fetus to adults (Kang et al., 2011; Spiers et al., 2015; Jaffe et al., 2016), the trajectory of molecular signatures of peripheral tissues could be captured by extraction of tissues *in vivo* throughout the life span after birth (Richmond et al., 2015; Urdinguio et al., 2016; Walton et al., 2017), and the trajectories of neuronal and brain based phenotypes could be measured by non-invasive brain imaging or signal techniques *in vivo* after birth (Asami et al., 2012; Li et al., 2012; Tamnes et al., 2013). For example, magnetoencephalography (MEG) can record neural activity at its natural millisecond temporal resolution. Magnetic resonance imaging (MRI) can measure brain morphometry and metabolite composition of brain tissue. Functional MRI can be used to probe brain activation responding to a specific task or during rest. Other techniques including electroencephalography (EEG), positron emission tomography, and near-infrared spectroscopy can also be used. Hereafter we refer all these techniques as neuroimaging techniques. More importantly, these neural and brain based phenotypes can be used to identify the neuronal underpinnings of human behavior, including cognition and symptoms, which ultimately lead to a disease diagnosis. One way to link the trajectories of molecular signatures, neural and brain based phenotypes, and human behavior is to evaluate the cross-tissue consistency of the information from easily accessible tissues. Approaches to systemically leverage the rich information collected across tissues and along the development trajectory could greatly assist in uncovering the disease pathogenesis and pathophysiology, beyond the identification of risk genetic mutations from genotype-phenotype association analyses.

### *In vitro* Cells and Neurons

Cell based approaches in general help elucidate the molecular and cellular basis of the disorders. A few neuronal cell models have been used to study psychiatric disorders, each with advantages and shortcomings as reviewed by Bray et al. (2012). Among them, the iPSN approach has gained the popularity due to its ability to capture a patient specific genetic background (i.e., patient derived iPSNs), and the potential to model specific cell types of interest, such as dopaminergic neurons (Robicsek et al., 2013) or GABAergic interneurons (Liu et al., 2013). The advances and challenges of iPSC/iPSN in SZ or general neuropsychiatric disorders have been summarized in Soliman et al. (2017) and Vadodaria et al. (2018). SZ patient iPSNs have been valuable to investigate the causal effects of SZ risk mutations via epigenetic (including open chromatin, DNA methylation, and histone modification) and transcriptomic signatures of iPSNs (Wen et al., 2014; Watmuff et al., 2016; Forrest et al., 2017), as well as neuronal physiology and morphology (Wen et al., 2014; Windrem et al., 2017). Forrest et al. showed that neuronal open chromatin regions in iPSNs are significantly enriched for SZ risk variants (Forrest et al., 2017), and prioritized ∼100 putatively functional risk SNPs through residing in open chromatin regions and near transcription factor binding sites. Furthermore, they demonstrated that one prioritized SNP, rs1198588, indeed regulates expression of miR-137 in neurons derived from iPSCs, altering dendritic complexity and synapse maturation. This epigenetic profile along with transcriptomic profiles from SZ patient derived iPSNs (Brennand et al., 2011, 2015) carry great potential to help identify and validate the causal effect of risk SNPs derived from GWAS.

### Postmortem Brain Tissues

Unlike iPSN studies that probe very early neural development mechanisms, studies of postmortem brains can cover a large age range from prenatal to adulthood. While studies on SZ patient postmortem brains (mostly adulthood) did not show obvious pathological anomalies, dysregulations of epigenetics and transcriptomics in selective brain regions/neurons and chromosome loci have been reported (Mistry et al., 2013; Chen C. et al., 2014; Numata et al., 2014; Wockner et al., 2014). However, it is difficult to disentangle the causative effects from the consequences of a lifetime illness or medication use. One approach to mitigate this problem is through the comparison with molecular impact of genetic risk mutations (Gibbs et al., 2010). For instance, SZ risk SNPs regulating epigenetics (such as meQTLs) (Numata et al., 2014; Hannon et al., 2016b) and transcriptomics (eQTLs) (Gamazon et al., 2013; Bacanu et al., 2014; GTEx-Consortium, 2015) in the brain have opened a new perspective to understand the functional impact of risk mutations. Two independent studies published at the same time consistently documented the effects of prevalent meQTL in both fetal and adult brains, and their significant associations with SZ risk (Hannon et al., 2016b; Jaffe et al., 2016). Hannon et al. (2016b) found that a majority (83%) of meQTLs in fetal brains were conserved in adult brains, and enriched for SZ risk, regulatory domains, and eQTLs. Moreover they presented an example on how to use co-localization information of meQTLs and risk SNPs to prioritize putative causal regions. Among these regions was the *AS3MT* locus in chromosome (chr.) 10, for which an independent group confirmed its causal effect (Li et al., 2016). Mutations in this region can alter expression of human-specific AS3MT isoform and BORCS7 in several cell lines and postmortem brains, and the expression of AS3MT isoform and BORCS7 were significantly higher in patients with SZ than healthy controls. Moreover DNA methylation in AS3MT was also significantly correlated with the expression of AS3MT isoform, indicating methylation might affect alterative AS3MT gene splicing (Li et al., 2016).

In addition, the temporal profiles of epigenetics and transcriptomics could shed insight into the disease developmental course. Jaffe and coworkers studied DNA methylation changes during different time periods. In the 10–25 years age range, roughly around the SZ disease onset time, DNA methylation changes were surprisingly not enriched for SZ risk. In contrast, methylation changes during prenatal-postnatal transition were widespread and prominent, and enriched for schizophrenia risk loci (Jaffe et al., 2016). Similarly, transcriptome profiles of human brain showed distinct and dynamic expression patterns for genes associated with SZ (Kang et al., 2011; Gulsuner et al., 2013). In a specific review of meQTLs in SZ (Hoffmann et al., 2016), the authors suggested that integrated comparison of brain methylomes along the development could help define the time window during which epigenomic dysregulation arises and how this intersects with any harmful exposure of mothers, unborn babies, and children. This viewpoint can also be applied to eQTLs and transcriptomes. This temporal information along with others (Brown and Derkits, 2010; Nielsen et al., 2014) lent further support to the neurodevelopmental origin of SZ (Birnbaum and Weinberger, 2017). Collectively, knowledge of the molecular signatures of postmortem brains would promote causal models for SZ risk mutations and disease progress models.

### Peripheral Tissues

Peripheral tissues such as blood and saliva have long been used to study indicators or biomarkers for the disease or treatment responses. Multifactorial features including epigenetics, and transcriptomics and proteomics as reviewed by Lai et al. are flourishing (Lai et al., 2016; Teroganova et al., 2016), with some holding the promise of potential utility for diagnostic purposes (Chan et al., 2015). Particularly, DNA methylation profiles have provided a promising capability to indicate diagnostic status (Aberg et al., 2014; Hannon et al., 2016a), symptoms (Liu et al., 2014), onset time (Melas et al., 2012), and environment insults (Aberg et al., 2014). Given that DNA methylation is a relatively new feature being investigated, some of the current findings suffer from inconsistency and lack of replicability (Jaffe and Kleinman, 2016) potentially due to cell composition, medications, substance use, and other environmental factors. In contrast, the meQTL findings in peripheral tissues provided relatively high consistency both across cohorts and across time points (Smith et al., 2014; Teh et al., 2014; Gaunt et al., 2016). Moreover, consistent meQTL effects across tissues (including brain, blood, saliva, and other tissues) has also been reported (Shi et al., 2014; Andrews et al., 2017; Lin et al., 2018b), and proposed to explain partially the methylation correspondence across tissue types (Hannon et al., 2016a; Andrews et al., 2017; Lin et al., 2018b). For instance, CpGs targeted by cross-tissue meQTLs showed high brain-blood methylation correlations compared to others, and an increase of meQTL effect size was linked to an increase of probability of targeted CpGs showing brain-blood correlations on methylation levels (Lin et al., 2018b). Similarly cross-tissue correlated gene expression and consistent eQTL regulations have also been discovered (Price et al., 2011; Torres et al., 2014). Both cross-tissue meQTLs and eQTLs showed relevance to SZ risks (Andrews et al., 2017; Lin et al., 2018b). In parallel, consistent cross-tissue (brain and blood) epigenetic responses to drug or stress have been reported (Abdolmaleky et al., 2015; Seifuddin et al., 2017), adding to the value of peripheral tissues in the study of neuropsychiatric disorders.

### Brain Based Phenotypes

Neuroimaging techniques provide non-invasive ways to measure brain or neural morphometry, activation, connectivity, and others. Features derived from *in vivo* brain imaging present intermediate phenotypes underlying human behavior from babies to older populations, which clearly are essential to studying SZ or any psychiatric disorder. A relatively new research field, imaging-genomics (Meyer-Lindenberg, 2010; Liu and Calhoun, 2014) aims at the associations between genetic variants and brain based intermediate phenotypes. Recent approaches and extensions of imaging genomics have just been reviewed (Bogdan et al., 2017; Mufford et al., 2017), highlighting future directions by which the field can move forward to shed light on brain disorders. Here we want to highlight the importance of multilevel multimodal analyses in which data of epigenetic, transcriptome and brain imaging/signals are integrated (Liu J. et al., 2015; Richiardi et al., 2015; Romme et al., 2017; Lin et al., 2018a). For example, one pioneering work to bridge the gap between transcriptome of postmortem brains and *in vivo* brain activity measured by fMRI (Richiardi et al., 2015) compared high-resolution gene expression profiles from cortical areas provided by the Allen Brain Institute and four well-defined functional brain networks at resting state, and found a set of genes whose expression significantly associated with these functional networks. This work suggested a correlated gene expression underlying synchronous activity in brain networks (Richiardi et al., 2015). Following this line of research, Romme and colleagues conducted a SZ specific study (Romme et al., 2017), where cortical transcriptional profiles of 43 SZ risk genes selected from GWAS were compared with cortical white matter fiber connectivity derived from diffusion-weighted imaging (DWI) in both SZ patients and controls. They found transcriptional profiles of risk genes significantly correlated with patient-control differential white matter disconnectivity pattern, and further confirmed this connection was specific to SZ patients as it was not seen in bipolar patients (Romme et al., 2017). A more detailed review of gene expression and brain function can be found in Konopka (2017).

On the other hand, epigenetic profiles of postmortem brains are, to date, limited to selective brain regions; there is no high resolution epigenetic profile of whole brain yet. Most studies to link epigenetic with brain phenotypes *in vivo* have to leverage peripheral tissue data. One of the earliest imaging-epigenetic studies linked the DNA methylation of one site (rs4680 Val) in the *COMT* gene in peripheral blood mononuclear cells negatively with lifetime stress and brain activity in prefrontal cortex, suggesting greater stress and lower methylation were related to reduced cortical efficiency in healthy participants (Ursini et al., 2011). Complementary to this study, we investigated *MB-COMT* promoter methylation in patients with SZ (Walton et al., 2014). Our results showed methylation in the promoter region of *MB-COMT* positively associated with brain activity in the left prefrontal cortex, where patients had reduced *MB-COMT* promoter methylation consistent with promoter hypomethylation of *MB-COMT* observed in postmortem brains (Abdolmaleky et al., 2006). Both studies consistently demonstrated how changes in DNA methylation of *COMT* in peripheral tissues were related to brain function in prefrontal cortex. DNA methylation patterns have also been associated with brain structure deficits in SZ patients (Liu J. et al., 2015) among other disorders (Shelton et al., 2016; Won et al., 2016). We expect to see more studies of epigenetics in relation to brain based phenotypes with increased availability of epigenetic data in both postmortem brain and peripheral tissues.

As illustrated above, knowledge gained from different tissues each presents a specific aspect or state of the disease etiology or pathophysiology, which are complementary to one another. iPSN studies demonstrated the very early impact of risk genetics at a rather homogenous cellular level. Postmortem brain studies can reflect the footprint of the disease developmental course in an organ of interest. Peripheral tissue studies can capture the live characteristics of diseases, including end products of genetics, environment, and their interactions, in an indirect fashion. Brain imaging records *in vivo* brain activation, brain structure and other brain dynamic features as an intact organ that greatly facilitates our understanding on human behavior and abnormalities associated in psychiatric disorders. Multilevel multimodal studies attempting to leverage information derived from each tissue type should be able to draw a more complete picture of functional impact of genetic risks during the disease development and manifestation.

### Multimodal Analyses

Multimodal analyses have been implemented more frequently for data collected from the same tissue. For instance, multimodal imaging data including structural MRI, DWI, fMRI, EEG, and MEG have all been analyzed jointly to leverage strength of each data modality and provide more in-depth look of brain. More detailed advantages and challenges of multimodal imaging analyses can be seen in several reviews (Sui et al., 2012; Liu S. et al., 2015; Pearlson et al., 2015; Calhoun and Sui, 2016). Meanwhile, integrated multi-omics studies are also increasing with the advance of techniques to obtain genomics, epigenomics, transcriptomics, proteomics, etc. Ritchie et al. provided a very nice systematic review on models to integrate multi-omics data (Ritchie et al., 2015). While most of the published studies focused on cancer, studies of neuropsychiatric disorders are catching up. Early works on methylome and transcriptome of postmortem brains in SZ showed very limited gene expression and epigenetic concordances in relation to SZ (Chen C. et al., 2014), but as proof-of-concept represented a novel approach for identifying candidate genetic factors in the etiology and pathology of neuropsychiatric disorders. A recent study in peripheral blood mononuclear cells of youth were able to identify 43 risk genes through a combined transcriptome and methylome analysis that discriminated bipolar patients and high-risk youth from controls (Fries et al., 2017). The authors went further and verified the role of DNA methylation in modulating gene expression through cell line treatment of a DNA methyltransferase inhibitor.

### Multilevel Multimodal Analyses

**FIGURE 2:**
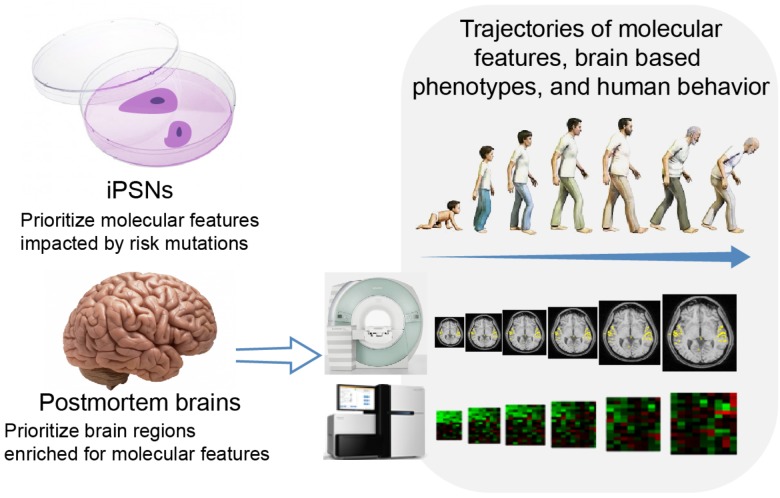
A hypothetical example of integration information across tissue types.

Multilevel multimodal analyses are beyond the aforementioned multimodal studies, which are limited to data modalities acquired from the same tissue type. As we indicated before, integration of information across tissues might put complementary information from different spatiotemporal points of the disease development course together. A hypothetical example, as illustrated in **Figure 2**, might be integration of molecular signatures of iPSNs and postmortem brains, temporal-spatial dynamics of postmortem brains, trajectories of features from blood, and brain *in vivo* in a multilevel model. First, epigenetic/transcriptomic features of iPSNs could be used to prioritize functional risk mutations as described in Forrest et al. (2017). The molecular features impacted by risk mutations in iPSNs could then be compared with that in postmortem brains to identify the brain regions of interest as reported in Negi and Guda (2017) and Romme et al. (2017). The dynamics of selected molecular features in selected regions of brain could be traced in postmortem brains in a population based manner as shown in Kang et al. (2011). And with cross-tissue eQTL/meQTL as the bridge the trajectory of the molecular features of the counterpart derived from blood could be monitored *in vivo*. Here data from healthy controls would provide a baseline of information across tissue types. Finally, the trajectories of blood features, brain based phenotypes and ultimately human behavior in a patient group could be assessed in a longitudinal fashion and then be analyzed along the life span. Data from patients would provide information on the disease progress. Even in view of the limitations and assumptions for methodological implementation, such an analysis in principle would potentially identify causal risk mutations and their impacts on pathogenesis and pathophysiology.

Toward this direction, we have recently reported SZ risk SNPs in chr. 6p22.1 linked to gray matter reduction in SZ patients (Chen et al., 2018). These SNPs also demonstrated strong regulatory effects. For example, SNP rs213240 modulated DNA methylation levels of both postmortem brain and saliva of a CpG set, of which one CpG site cg23266546 was hypermethylated in postmortem brain in SZ patients, indicating the T allele as the causal risk. Another site cg26335602 measured in saliva showed a significant positive association with gray matter volumes derived from imaging *in vivo*, identifying the T allele as the causal risk. SNP rs213240 also regulated gene expression of *ZKSCAN3* in postmortem brain, which showed a significant downregulation in SZ patients, indicating allele T is causal risk. Notably, risk alleles inferred from *in vivo* gray matter reduction were all consistent with those inferred from dysregulation of postmortem brain DNA methylation and gene expression, and dysregulation of saliva DNA methylation. This cross-cohort, cross-tissue convergence supports regulatory mechanisms of risk SNPs in 6p22.1 to exhibit the effects on brain and behavior.

We also investigated a mediation model of SZ risk meQTLs (risk SNPs are meQTLs) which regulate DNA methylation and lead to brain structure change in SZ patients. Genomic DNA methylation in blood and saliva was obtained; their genetic regulation effect and relation to brain structure were analyzed (Lin et al., 2018a). SNPs in SZ risk regions implicated from GWAS demonstrated consistent regulation on DNA methylation in blood and saliva in two highlighted regions: chr10 ARL3-AS3MT-NT5C2, and chr3 NT5DC2-INT1-INT4. These regions’ meQTL regulation effect in brain was reported elsewhere (Hannon et al., 2016b; Jaffe et al., 2016). Peripheral DNA methylation in these two regions was associated with gray matter variation and showed significant mediation effects between risk SNPs and gray matter reduction. The strongest and most prevalent association between methylation and gray matter was from *NOSIP* gene, which functionally inhibits the activity of nitric oxide synthase and reduces nitric oxide production, to frontal, temporal, and occipital regions, where SZ demonstrated most gray matter reduction (Gupta et al., 2014). Overall, SZ patient differences were most prominent in brain structure, then DNA methylation, and then SNPs. Consistent results from blood and saliva support cross-tissue genetic regulation on DNA methylation and methylation from peripheral tissues being able to indicate brain status. Together, these studies showed the importance of mining multi-level information in the study of schizophrenia.

To date, very few studies have investigated multilevel multimodal data. Including broadly multimodal analyses, the analytical approaches implemented can be roughly grouped into two categories: (1) parallel integration where data from different modalities are treated equally and in parallel, and (2) model-based integration where a specific model for the relationship among different modalities is proposed and tested. For parallel integration, analytical methods such as multimodal canonical component analysis (Nielsen, 2002) or N-way parallel ICA (Vergara et al., 2014) as reviewed in Sui et al. (2012); Liu and Calhoun (2014); and Ritchie et al. (2015) can be used to directly mine the data of different tissues/levels. Another approach for parallel integration is to analyze data from each modality separately first, and then integrate the derived information such as significance and effect size at the second level in a meta-analysis fashion (Jia et al., 2018). For the model-based integration, various methods from basic statistical tests to complex data factorization are usually combined to fit into the desired model, as shown in several highlighted works (Richiardi et al., 2015; Chen et al., 2018; Lin et al., 2018a). It can also be a simple cascade model where outputs derived from one level in the hierarchy of the biology are fed into another, such as DNA sequence to epigenetic elements, to gene expression, to protein and cells, and to organs, as represented partially in Li et al. (2016) and our submitted work linking a circular RNA to neuronal function and cognition. By all means, studies integrating multilevel multimodal data are just emerging, and the need for more analytical approaches is becoming and will continue to be pressing along the development of this specific research focus.

### Challenges and Caveats

Challenges and caveats should be carefully taken into account in any future multimodal cross-tissue studies. Tissue specificity is clearly an important issue. It is well known that epigenetics and transcriptomics are tissue specific, which is essential for stem cells differentiating into specific functional tissue lineages. Understanding both tissue specific and cross-tissue characteristics of molecular features, consistent, complementary, or differential genetic–epigenetic–transcriptomic-proteomic mechanisms across different tissues all can advance our understanding of diseases pathogenesis. To date, we have very limited knowledge about across tissues properties, and leveraging such limited knowledge (e.g., cross-tissue eQTL/meQTL) into the cross-tissue design poses a challenge.

There are also caveats associated with each individual tissue. Currently iPSN techniques are still in the early phase. Different protocols and culture conditions for differentiating neurons induce a large amount of variability in the measurements. Schwartzentruber and colleagues studied 123 differentiations of iPSCs and suggested typically iPSC lines from 20 to 80 unrelated individuals are needed for analyses of genetic variants with allelic fold change between 1.5 and 2 fold (Schwartzentruber et al., 2018). Epigenetics and transcriptomics in postmortem brains are affected by agonal and postmortem factors (Tomita et al., 2004). Proper controls or corrections to minimize these factors are always required. Another challenge to use data from postmortem brains is availability. To date, the Allen Institute provides high resolution transcriptome profiles of six brains of healthy subjects on which most of studies have been based. There is no similar data on epigenetics yet. Also, data on patient groups and data along the life course are only available on selected brain regions. Careful designs are necessary to leverage current information, in hope that in the near future more data will be available. Brain imaging measures are affected by equipments and parameter settings (Chen J. et al., 2014). Particularly in large collaborative studies of multiple centers, proper controls on the data assessments and analytic corrections to mitigate the effects are important. The ENGIMA project is a successful example of how to conduct large collaborative imaging-genetic studies (Thompson et al., 2014; Hibar et al., 2015).

Confounding effects such as those of medication on epigenetics (Swathy and Banerjee, 2017), transcriptome (Crespo-Facorro et al., 2014), and brain structure and activation (Scherk and Falkai, 2006; Borgwardt et al., 2009; Smieskova et al., 2009) are well documented in patient groups. Studies on cross-tissue data need to recognize that interrelationship could be superimposed by confounding effects. Last but not the least, analytical approaches to integrate such data in conjunction with sufficient statistical power poses another layer of challenge. So far several aforementioned approaches have been used for multilevel multimodal analyses, but the greatest need is to systemically leverage cross-tissue complementary power for which we expect that more tissue related knowledge needs to be modeled in addition to data modalities. Despite these challenges and caveats there is great potential for the use of this systemic integration approach.

### Summary

Overall future studies could integrate information derived from iPSNs, such as genomic open chromatin profile and transcriptome profile, information derived from postmortem brain, such as high resolution whole brain transcriptome map, information from peripheral tissues indicating disease progress as an outcome of interactions between genetic and environment, together with information from brain imaging and behavior, ultimately linking to diseases. Such multilevel multimodal approaches, which we expect to be implemented more frequently in the future, will pave the way to go beyond genotype-phenotype association studies. Of course other fundamental approaches are also of great importance, including animal models, cell line manipulations, gene editing, which are beyond this selective review. Also, other molecular signatures such as RNA isoforms and splicing QTLs (Takata et al., 2017), proteomic features, and cellular features are all important, although were not discussed here. Other types of tissues may also be used, such as placenta for which the transcriptional profiles are suggested to drive the interaction between early-life-complication and genetic risks for the development of SZ (Ursini et al., 2018). The specificity of this tissue type indeed helps to elucidate the largely unknown interaction between early-life environment and genetics on the etiology of diseases. The general principle of integrating multiple lines of evidence is applicable to other modalities and to other psychiatric disorders beyond SZ. In our opinion, we are entering into the post GWAS era.

## Author Contributions

JL has designed this study and drafted the manuscript. All authors contributed to reviewing and editing the final manuscript.

## Conflict of Interest Statement

The authors declare that the research was conducted in the absence of any commercial or financial relationships that could be construed as a potential conflict of interest.
